# The Improvement of Energy Storage Performance by Sucrose-Derived Carbon Foams via Incorporating Nitrogen Atoms

**DOI:** 10.3390/nano11030760

**Published:** 2021-03-17

**Authors:** Malgorzata Skorupska, Piotr Kamedulski, Jerzy P. Lukaszewicz, Anna Ilnicka

**Affiliations:** 1 Faculty of Chemistry, Nicolaus Copernicus University in Torun, Gagarina 7, 87-100 Torun, Poland; m.skorupska@doktorant.umk.pl (M.S.); pkamedulski@umk.pl (P.K.); jerzy_lukaszewicz@o2.pl (J.P.L.); 2 Centre for Modern Interdisciplinary Technologies, Nicolaus Copernicus University in Torun, Wilenska 4, 87-100 Torun, Poland

**Keywords:** graphene, sucrose, chitosan, carbonization, carbon nanocomposite, microporous structure, surface functional groups

## Abstract

This paper addresses the problem of improving electrochemical energy storage with electrode materials obtained from common raw ingredients in a facile synthesis. In this study, we present a simple, one-pot route of synthesizing microporous carbon via a very fast reaction of sucrose and graphene (carbon source), chitosan (carbon and nitrogen source), and H_3_PO_4_. Porous carbons were successfully produced during high temperature carbonization, using nitrogen as a shielding gas. Samples were characterized using X-ray powder diffractometry, elemental analysis, N_2_ adsorption-desorption measurements, scanning electron microscopy, and Raman spectroscopy. The developed carbon material possessed a high surface area, up to 1313 m^2^ g^−1^, with no chemical or physical activators used in the process. The structural parameters of the microporous carbons varied depending on the ratio of reagents and mass composition. Samples were prepared both with and without chitosan. The present synthesis route has the advantages of being a single-step approach and only involving low-cost and environmentally friendly sources of carbon. More importantly, microporous carbon was prepared without any activators and potentially offers great application in supercapacitors. Cyclic voltammetry and constant current charge–discharge tests show that sucrose-based porous carbons show excellent electrochemical performance with a specific capacitance of up to 143 F g^−1^ at a current density of 1 A g^−1^ in a 6 M KOH electrolyte.

## 1. Introduction

Fabrication of electrochemical energy storage devices (batteries and supercapacitors) greatly relies on carbon-based electrodes. Therefore, improving the efficiency of these devices will rely on new findings in the area of carbon electrode material synthesis. Solutions encompassing a wide accessibility of raw materials, acceptable market price, and facile processing are in high demand due to the need of eventual mass production. Natural materials such as biomass and carbohydrates (e.g., glucose, fructose, lactose, and cellulose), which have natural reserves, are being extensively studied as a source of carbon-based materials. Due to its uniform structure, very low price, local availability, and high chemical purity, sucrose is one of the most attractive carbon precursors. There are essentially two main approaches that can be applied to extracting carbon from sucrose, either a hydrothermal [1] or a heat-treatment process [2,3,4,5]. To improve the porous structure of sucrose-based carbon, a variety of activation methods have been developed, which use steam [6], CaCO_3_ [7], NaHCO_3_ [8], polyurethane as a template [9], CO_2_ gas [10], LiOH [11], NaOH [11,12,13], KOH [11,14,15], H_3_PO_4_, ZnCl_2_, SnCl_2_, or CaCl_2_ [16]. Another approach to synthesis is to activate sucrose mechanically [17,18,19]. The micropore volume in a sucrose-based structure may depend on dehydrating acids (sulphuric, hydrochloric, phosphoric, oxalic, acetic, citric, and tartaric acid) and the cationic component (Ca(NO_3_)_2_, (NH4)_2_SO_4_, (NH_4_)_2_CO_3_, NH_4_) used during synthesis [20]. Using the carbonization of a triblock copolymer (P123), silica, and sucrose composite, highly ordered mesoporous carbon has been generated [21,22].

Electrode materials based on activated carbon are widely used in supercapacitors [23,24] and Li-ion batteries [25,26]. In our previous research, we found that pyrrolic-N (N-5), pyridinic-N (N-6), and quaternary-N (N-Q) groups, active in catalytic reactions, are present in carbon obtained using chitin, chitosan, green algae, or amino acids as a source [27,28,29]. Therefore, using one of these precursors during the synthesis of sucrose-based carbon for supercapacitors should augment energy storage performance. Recently, the electrochemical properties of sucrose-derived carbon were improved by doping with N, F, and B, utilizing H_3_BO_3_ or NH_4_F as heteroatom precursors [9]. Huang et al. prepared chemically activated carbon (with KOH) for electric double layer capacitors (EDLC) with a very modest specific capacitance of only ~40 F g^−1^ [30]. For porous carbon materials prepared by Guo et al. via high-temperature pyrolysis, the capacitance equaled 232 F g^−1^ at a current density of 0.1 A g^−1^ [31]. In another study, Subramanian et al. used sucrose pre-treated with ammonia nitrate as the carbon source to prepare a porous carbon with a specific capacitance of 232 F g^−1^ [32]. Silicon nanowires coated with sucrose-derived carbon were prepared for supercapacitor application [33] as well. The disadvantages of the mainstream electrode material production methods are the often multiple-step procedures, environmentally unfriendly reagents, and the presence of metals or metal oxides in the materials’ structure.

Recognizing the applicability of sucrose in N-doped carbon electrode materials was the main motivation of the current study. To the best of the authors’ knowledge, so far, there have been no reports of a one-pot synthesis of porous carbon foam from materials such as hydrocarbons and graphene, without using an activation agent. In this paper, different carbon and nitrogen contents were prepared in a carbonization process using sucrose and graphene as carbon sources and chitosan as a carbon and nitrogen source; their structural and electrochemical properties were evaluated. By changing the ratio of reagents, well-developed micro or mesoporous structures were obtained. The use of chitosan caused nitrogen groups to be introduced into the porous foam carbon structure.

## 2. Materials and Methods

### 2.1. Materials

Graphene nanoplatelets (750 m^2^ g^−1^) and chitosan were purchased from Sigma Aldrich (branch in Poland). Sucrose was purchased from Krajowa Spolka Cukrowa S.A. (Torun, Poland). Other reagents, i.e., H_3_PO_4_ and CH_3_COOH, were purchased from POCH (Gliwice, Poland).

### 2.2. Preparation of Carbon Foams

Carbon foams were synthesized in one of two ways. In the first path, to 3 or 5 g of sucrose, 1 or 3 g of graphene nanoplatelets was added and mixed well. In the second, to 3 or 5 g of sucrose, 1 or 3 g of chitosan was added, previously dissolved in 10 mL of a 1% CH_3_COOH solution, then mixed. In the next step, the mixtures were treated with 3 or 4.5 mL of H_3_PO_4_ at a 3:1 and 5:3 ratio of reagents, respectively. Samples were then heated on a double electric cooker at temperatures in the range of 100 to 400 °C. After this, materials were carbonized in a N_2_ atmosphere at a heating rate of 10 °C min^−1^ until 900 °C was reached. This temperature was maintained for 1h. The process was carried out in a tubular furnace (Thermolyne F21100) (NIST, Gaithersburg, MD, USA). Further in the text, the mixture of sucrose (S) and chitosan (CS) used during synthesis is denoted as SCS. The mixture of sucrose (S) and graphene nanoplatelets (GF) is denoted as SGF. The mass ratio (R) of reagents used, S:CS and S:GF, was either 3:1 or 5:3. The samples in general are indicated as SCS-R (SCS-3:1, SCS-5:3) and SGF-R (SGF-3:1, SGF-5:3).

### 2.3. Structure Characterization

The morphology of the obtained samples was determined using a scanning electron microscope (SEM 1430 VP, LEO Electron Microscopy Ltd., Oberkochen, Germany) operating at 30 kV. The porous structure of the samples was analyzed via a nitrogen adsorption experiment at 77 K, using an automatic adsorption instrument, ASAP 2020 Plus (Micromeritics, Norcross, GA, USA). Before the analysis, obtained carbons were outgassed in a vacuum at 200 °C for 24 h. The samples’ surface areas were calculated using the Brunauer–Emmett–Teller (BET) equation, and the pore size distributions were calculated using the nonlocalized density functional theory (NLDFT) method. The elemental composition of the materials was analyzed by means of a combustion elemental analyzer (Vario CHN, Elementar Analysensysteme GmbH, Langenselbold, Germany). Raman spectra were obtained by a Renishaw InVia Raman analyzer (laser wavelength 532 nm, Renishaw Company, Gloucestershire, UK). X-ray photoelectron spectroscopy (XPS, PHI5000 VersaProbe II Scanning XPS Microprobe, Chigasaki, Japan) measurements were performed using a monochromatic Al Kα X-ray source. Survey spectra were recorded for all samples in the energy range of 0 to 1300 eV with a 0.5 eV step, while high-resolution spectra were recorded with a 0.1 eV step.

### 2.4. Electrochemical Measurements

The electrochemical performance of carbon samples was studied using cyclic voltammetry (CV) curves, galvanostatic charge–discharge (GCD) cycling, and electrochemical impedance spectroscopy (EIS) plots for a two-electrode system. Electrodes prepared for electrochemical measurements were well-mixed active materials, conductive carbon black (C-140), and polytetrafluoroethylene (PTFE) at a mass ratio of 17:2:1. These, at a diameter of 9.25 mm, were dried in a vacuum oven at 100 °C for 24 h. Electrochemical capacitors were built using two carbon electrodes with comparable mass (4–5 mg). Cyclic voltammetry and galvanostatic charge–discharge data were collected using a potentiostat–galvanostat (PGSTAT128N, Autolab) (Metrohm Autolab B.V., Utrecht, The Netherlands). Electrochemical investigations were performed in 6 M KOH aqueous electrolyte. Cyclic voltammograms were recorded at a scan rate between 5 and 200 mV s^−1^, in the potential range of 0 to 0.9 V. Charging-discharging was performed at various current densities, from 0.1 to 40 mA g^−1^. Electrochemical impedance was measured in a frequency range of 10^−1^ to 10^5^ Hz; the amplitude was 10 mV. Two electrodes of similar mass were used in a symmetrical two-electrode supercapacitor. The specific capacitance C_s_ (F g^−1^) (Equation (1)), energy density E (Wh kg^−1^) (Equation (2)), and power density P (W kg^−1^) (Equation (3)) were calculated based on the following equations:

C_s_ = I × Δt/(m × ΔV) (1)

E = (C × ΔV^2^)/(8 × 3.6) (2)

P = (E × 3600)/Δt(3)

where I is the charge and discharge current (A), Δt is the discharge time (s), ΔV is the potential change within the discharge time (V), and m is the total mass of active materials in both electrodes.

## 3. Results and Discussion

### 3.1. Characterization of Carbon Materials

New carbon-based materials have been synthesized and characterized according to the specific underlying motivation: recognizing the applicability of sucrose for N-doped carbon electrode materials. Particular attention was paid to the insertion of nitrogen atoms, commonly regarded as the way to improve electric charge storage. Moreover, the authors searched for the most facile synthesis route. Characterization included key features like surface area and pore structure, microscopic structure and morphology, bulk and surface elemental composition, as well as collection of a complete set of electrochemical profiles.

**Figure 1:**
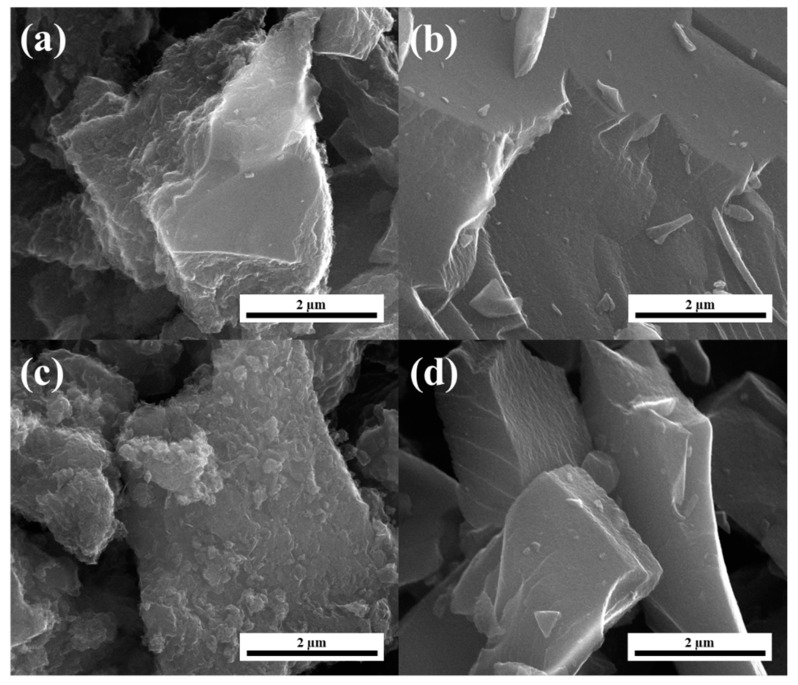
SEM images of the (**a**) mixture of sucrose and graphene nanoplatelets with a 3:1 ratio (SGF-3:1), (**b**) mixture of sucrose and chitosan with a 3:1 ratio (SCS-3:1), (**c**) mixture of sucrose and graphene nanoplatelets with a 5:3 ratio (SGF-5:3), and (**d**) mixture of sucrose and chitosan with a 5:3 ratio (SCS-5:3) sample.

Figure 1 shows scanning electron micrographs for samples obtained from sucrose with two different modifications—chitosan and graphene. The pores developed under chitosan influence are interdigitated with each other to form a three-dimensional (3D), interconnected frame structure, the morphology of the carbon materials being sensitive to chitosan use. This special structure enables the pores inside the material to communicate with each other and allows the electrolyte to penetrate the electrode material more smoothly, accelerate the transport of electrolyte ions, reduce the “traffic blockage” in the ion transport process, and thus improve capacitance performance [34,35]. The SCS-R samples (Figure 1a,c) and SGF-R samples (Figure 1b,d) exhibit differences in morphology. A smooth surface of carbon can be attributed to a lower specific surface area (S_BET_) when comparing SGF-3:1 and SGF-5:3 to SCS-3:1 and SCS-5:3 carbon, respectively, as described in Table 1.

**Figure 2:**
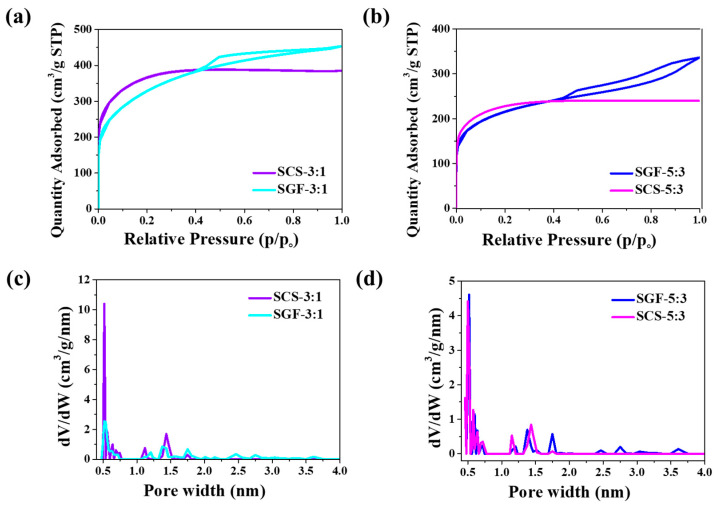
(**a**,**b**) N_2_ adsorption–desorption isotherms at 77 K; (**c**,**d**) nonlocalized density functional theory (NLDFT) pore size distribution obtained from adsorption branches of N_2_ in the SGF-R and SCS-R samples.

The nitrogen adsorption-desorption isotherms of the carbon prepared from sucrose possess the appropriate distinct shape of carbon obtained with graphene or chitosan (Figure 2a,b). The shapes of isotherms belong to type I and IV, according to the International Union of Pure and Applied Chemistry (IUPAC) classification [36]. Using sucrose and graphene or chitosan primarily creates micropores in the structure. As listed in Table 1, the N_2_ adsorption experiments indicate that the highest BET surface area is 1111 m^2^ g^−1^ for SGF-3:1 and 1313 m^2^ g^−1^ for SCS-3:1. Interestingly, the surface areas of samples obtained at a 5:3 weight ratio (lower sucrose content) are lower than those of samples at the 3:1 ratio. The specific surface areas for samples SGF-5:3 and SCS-5:3 are 771 and 841 m^2^ g^−1^, respectively. The total pore volume is given in the range of 0.37 to 0.70 cm^3^ g^−1^. Figure 2c,d provides pore size distribution as obtained using the NLDFT; size distribution appears to be quite narrow. The two different mass ratios of graphene to sucrose (3:1, 5:3) affect pore size. For samples SCS-3:1 and SGF-3:1, micropore size ranges from 0.5 to 1.8 nm. Samples SCS-5:1 and SGF-5:3 show micropores and small mesopores, up to 3.75 nm. The pore size distribution shows that sucrose-derived carbon obtained without any activator would be a suitable electrode material for supercapacitors [37,38].

Elemental composition results are given in Table 1. Influence of graphene and chitosan on the carbon and nitrogen content is observable. Use of graphene (GR) caused carbon content to increase to 79.72–90.18 wt.%. For the samples obtained with chitosan, carbon content decreased to 62.49–67.79 wt.%. Generally, higher carbon content is reported for samples synthesized with a higher amount of sucrose. The content of nitrogen for samples obtained with chitosan is 1.97 wt.% for SCS-3:1 and 2.13 wt.% for SCS-5:3, while the nitrogen content for samples obtained with graphene significantly decreased and is only 0.60 wt.% for sample SGF-3:1 and 0.3 wt.% for SGF-5:3. The reason behind the low nitrogen content is a lack of nitrogen precursors.

**Figure 3:**
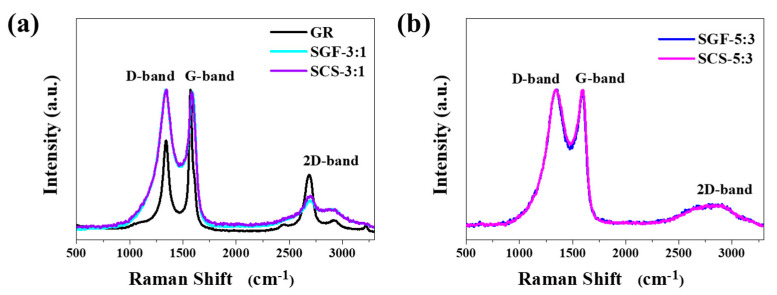
Raman spectra of (**a**) graphene, SGF-3:1 and SCS-3:1, (**b**) SGF-5:3 and SCS-5:3 sample.

Raman spectroscopy was used to characterize the graphitization degree of carbon materials [39,40]. Figure 3 shows Raman spectra for the carbon materials synthesized from sucrose and chitosan or graphene. In all cases, Raman spectra exhibit the D-band, G-band, and 2D-band at a shift of ~1340 cm^−1^, ~1580 cm^−1^, and ~2700 cm^−1^, respectively. The intensity ratio of the D- and G-bands determines the level of graphitization [14,41,42]. The materials here show a disordered location of graphene with the intensity of the D-band and G-band (I_D_/I_G_) at 1 (Table 2). The lack of significant differences in the I_D_/I_G_ ratio confirms that using sucrose causes defects in the carbon structure.

However, materials synthesized from ingredients in a 3:1 ratio have a 2D-band which is more pronounced. As the 2D-band decreases, the number of graphene structure defects increases, which is observable for series 5:3. Thus, a transition from a few-layer graphene structure to an amorphous carbon material can be seen [43,44]. A higher number of overlapping graphene layers is observed, and therefore, relative intensities of the 2D-band and G-band (I_2D_/I_G_) are low, around 0.2.

**Figure 4:**
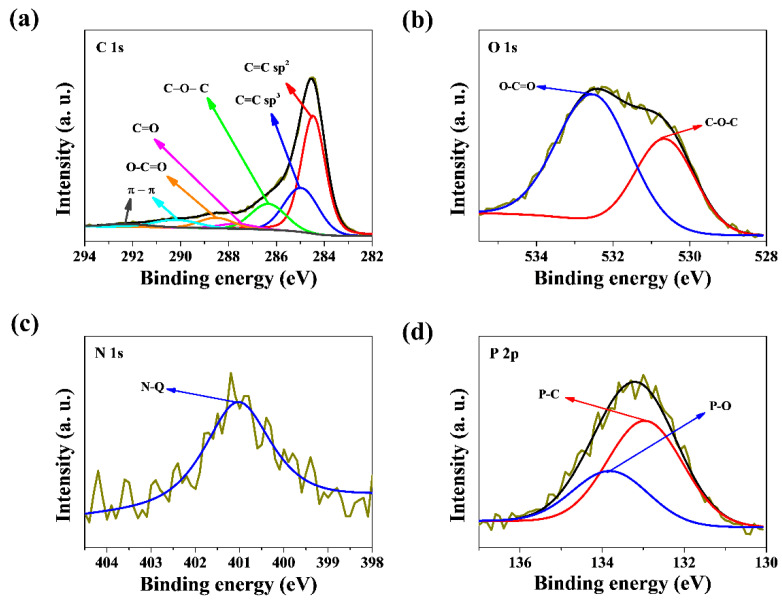
High-resolution XPS spectra: (**a**) C1s; (**b**) O1s, (**c**) N1s, and (**d**) P2p for the SCS-3:1 sample.

The porous carbon material’s surface element composition and chemical states of atoms were analyzed and characterized by XPS. In order to understand the chemical state of carbon, oxygen, and nitrogen atoms, XPS was recorded for samples SGF-3:1 and SCS-3:1; the results are presented in Figure 4.

The C1s spectrum is shown in Figure 4a, where two main characteristic peaks can be fitted: the peak with the strongest intensity at 284.6 eV, which corresponds to the presence of sp^2^ C=C bonds, and a characteristic peak at 285.0 eV, which corresponds to sp^3^ C-C bonds [45,46,47]. The C1s spectrum of samples was also deconvoluted and assigned to C-O-C bonds (286.3 eV), C=O or O–C–O bonds (287.7 eV), O–C=O bond (288.6 eV), and π-π (289.6 eV and 292.1 eV) [48]. The spectrum of O1s is shown in Figure 4b, where two main characteristic peaks can be fitted: the most important peak at 532.0 eV, corresponding to the C–O–C group [49], and the peak at 533.3 eV, corresponding to the O−C=O group [50]. The presence of oxygen and the corresponding functional groups can produce pseudo capacitor-induced currents and improve the wettability of the electrode material’s surface. This increases the amount of surface charge storage, thus helping to increase the energy density of the supercapacitor. XPS analysis evidenced the presence of nitrogen groups in the SGF-3:1 and SCS-3:1 sucrose-based foams. The results reveal that different samples present a different number of nitrogen heteroatoms. SGF-3:1 shows 0.9% at., while SCS-3:1 shows a higher nitrogen content of 2.1% at. In the case of SGF-3:1, there was no peak observed for nitrogen in the N1s region. Hence, it can be concluded that chitosan is responsible for the N-doping. One type of N-based species was identified in doped SCS-3:1, from the N1s XPS high-resolution spectrum (Figure 4c), assigned to quaternary-N (N-Q) at 400.7 eV [50,51,52,53]. It is important to point out that when N is incorporated into SCS-3:1, the relative contribution of N-species other than quaternary-N is increased compared to SGF-3:1. The N1s spectra imply that N atoms have partially substituted C atoms in the carbon lattice. The XPS spectrum of P2p (Figure 4d) in both samples presents two characteristic bonds of P–C and P–O at binding energies of 133.5 eV and 134.3 eV [54].

### 3.2. Electrochemical Performance

**Figure 5:**
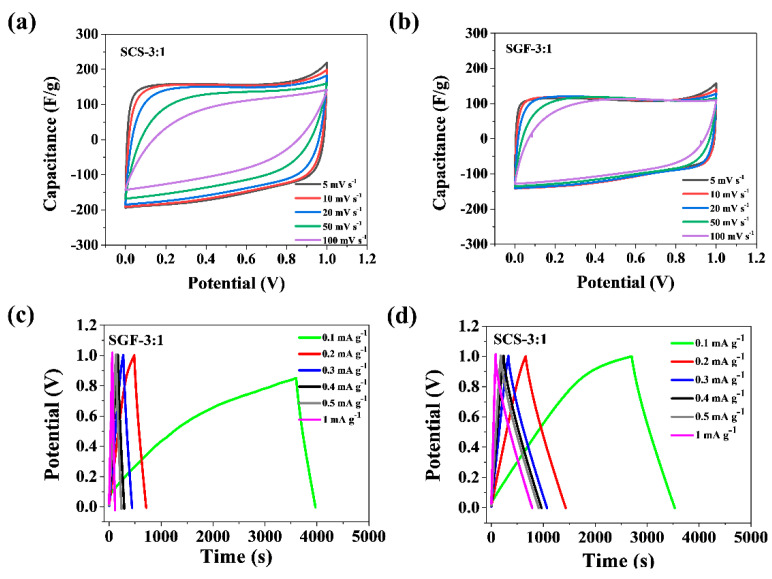
Electrochemical performance tests of the SCS-3:1 and SGF-3:1 samples: (**a**,**b**) cyclic voltammetry (CV) profiles at different scan rates ranging from 5 to 100 mV s^−1^; (**c**,**d**) galvanostatic charge-discharge curves.

Experimental results show that the carbon obtained from sucrose with no activator, only graphene and chitosan, possesses properties important for energy storage. The rectangular shape of the CV curve portrays fast re-organization of the electrical double layer at switching potentials and indicates fast ion transport, provided by an ideal EDLC charge storage mechanism [55,56,57]. A rectangular shape was observed in the curves of both the SCS-3:1 and the SGF-3:1 electrode, implying ideal double-layer capacitance behavior (Figure 5a,b). For sample SCS-3:1, the CV shape in a two-electrode cell is rectangular in all scan rates. The pore size distribution for series 3:1 is strictly microporous without any mesopores, and therefore, BET is higher for this series; these electrodes should possess the highest capacitance and the lowest resistance [58,59]. A key role in determining specific capacitance is played by the specific surface area and microporosity with a pore size larger than the ions of those electrolytes [60].

**Figure 6:**
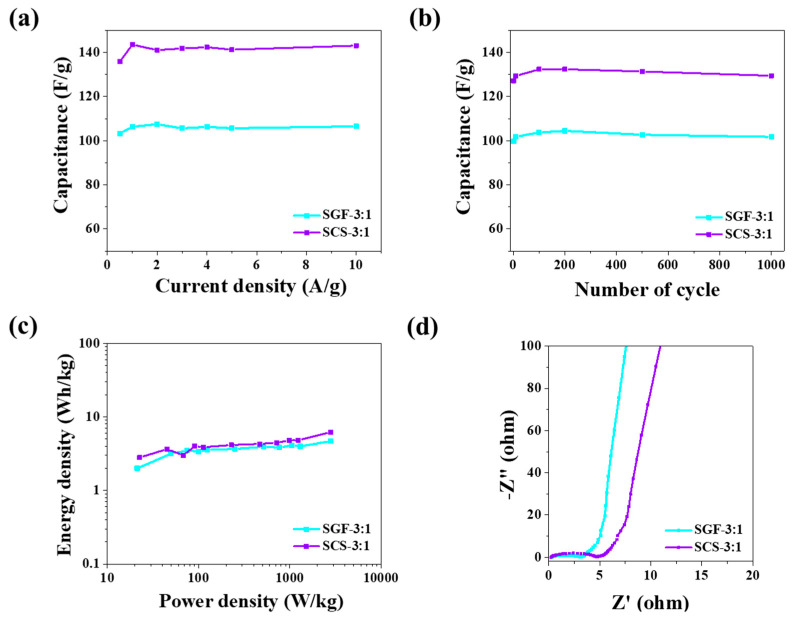
Electrochemical performance tests of the SCS-3:1 and SGF-3:1 samples: (**a**) specific capacitance as a function of current density; (**b**) specific capacitance as a function of cycle number; (**c**) specific capacitance as a function of power density; (**d**) Nyquist plot.

Specific capacitance (C_s_) was calculated using galvanostatic discharge curves (Figure 5c,d). Looking at the charge–discharge curves of the SCS-3:1 and SGF-3:1 samples, electrode SCS-3:1 shows the highest capacitance. When the current density equaled 1 A g^−1^, the specific capacitance was 143 F g^−1^ for electrode SCS-3:1, and when it equaled 2 A g^−1^, the specific capacitance was 107 F g^−1^ for SGF-3:1 (Figure 6a,b). When compared with other materials [61], those tested show a similar range of supercapacitor operation, in the range of 0 to 0.8 V. Naturally sourced materials’ specific capacity also trends similarly to the others, being within the range of 39–246 F^−1^. The difference between performance and capacity is caused by the presence of different functional groups on the surface, conductivity, wettability in electrolyte, and pore size, from which the diffusion barrier arises [50,51,52]. The N-Q functional groups identified in sample SCS-3:1 would benefit the enhancement of electrical conductivity, as well as capacitance, by interacting with anions in the alkaline electrolyte [50,62,63]. These microporous materials offer sufficient space to allow electrolyte ion transportation and thus improve performance in supercapacitors.

The specific capacity of the SGF-3:1 electrode increases from the initial value to 104 F g^−1^ due to self-activation, then after 200 discharge cycles, there is a slow decrease in the specific capacity value (97% (102%) retention after 1000 cycles). The SCS-3:1 electrode exhibits a similar disposition. The specific capacity is slowly increased to 132 F g^−1^ for 100 cycles, followed by a decrease in the specific capacity value, also at 97% (101%) retention after 1000 cycles (Figure 6b). These results indicate that microporous, sucrose-based carbon electrodes used in supercapacitors have good stability and capacitance retention. The plot presented in Figure 6c shows the relationship between energy densities and power densities, calculated from the charge–discharge measurements. Materials obtained with the proposed method show an energy density of 4.7 Wh kg^−1^ at a power density of 2.8 kW kg^−1^ for SGF-3:1 and an energy density of 6.2 Wh kg^−1^ at a power density of 2.8 kW kg^−1^ for SCS-3:1. At the same time, cellulose materials show an energy density of 13 Wh kg^−1^ at a power density of 27 kW kg^−1^. In another example of activated carbon nanotubes exhibiting an energy density of 1.0 Wh kg^−1^ at a power density of 14.4 kW kg^−1^ [64], the obtained foams show similar electrochemical properties.

Figure 6d shows the electrochemical impedance spectra of electrodes SCS-3:1 and SGF-3:1, in a frequency range of 10^−1^ to 10^5^ Hz. Nyquist plots of the samples showed typical features of porous electrodes with an irregular semicircle at high frequencies, a relatively short 45° Warburg region at high-medium frequencies, and a slash at low frequencies. At low frequencies, the SMC-800 electrode exhibits an almost vertical line, owing to the high specific surface area and pore size distribution of SCS-3:1, coupled with the electrolyte easily penetrating into its pores [65]. These results indicate that the SCS-3:1 electrode has an almost ideal capacitance at low frequencies and good frequency response performance in accordance with the capacity calculated from the galvanostatic charge–discharge curves [6,66].

## 4. Conclusions

The obtained results fully meet the expectations arising from the previously presented research motivation. The present synthesis procedure has the advantage of being a single-step approach and only involving the use of simple organic precursors (sucrose and chitosan as carbon and nitrogen sources), plus graphene and H_3_PO_4_ during carbonization. The samples exhibit a correlation between the amount of sucrose used and their textural properties, especially surface area and pore volume. When the ratio of graphene to sucrose is 3:1, the BET is higher. The SGF-R and SCS-R samples show a microporous structure with small mesopores below 4 nm. The sucrose-based carbon SCS-3:1 is a combination of sucrose and chitosan (carbon and nitrogen source) with the highest specific surface area of 1313 m^2^ g^−1^. Cyclic voltammetry (CV), electrochemical impedance spectroscopy (EIS), and galvanostatic charge–discharge cycle tests have been applied to investigate the capacitive performance of the SGF-R and SCS-R electrodes, in 6 M KOH, at room temperature. Electrochemical test results demonstrate that when the ratio of graphene and sucrose is equal to 3:1, the composite cathode for supercapacitors has a more stable performance than other samples. The SGF-R and SCS-R electrodes in 6 M KOH show specific capacitance in excess of 143 F g^−1^ at room temperature.

## Figures and Tables

**Table 1:** Elemental composition and textural parameters of the SGF-R and SCS-R samples.

Sample	Elemental Content (wt%)			S_BET_^1^ (m^2^ g^−1^)	V_t_^2^ (cm^3^ g^−1^)	V_mi_^3^ (cm^3^ g^−1^)	V_me_^4^ (cm^3^ g^−1^)
	C	H	N				
GR	87.32	0.90	0.72	750	1.00	0.13	0.87
SGF-3:1	90.18	0.71	0.60	1111	0.70	0.11	0.59
SCS-3:1	67.79	1.54	1.97	1313	0.60	0.13	0.47
SGF-5:3	79.72	1.13	0.30	771	0.52	0.20	0.32
SCS-5:3	62.49	1.97	2.13	841	0.37	0.30	0.07

^1^ S_BET_—total specific surface area, ^2^ V_t_—volume of total pores; ^3^ V_mi_—volume of micropores; ^4^ V_me_—volume of mesopores.

**Table 2:** Fitting results of the Raman spectra for graphene (GR), SGF-R, and SCS-R samples.

Sample	I_D_	Raman Shift (cm^−1^)	I_G_	Raman Shift (cm^−1^)	I_2D_	Raman Shift (cm^−1^)	I_D_/I_G_	I_2D_/I_G_
GR	0.64	1341	1	1570	0.4	2686	0.64	0.4
SGF-3:1	1	1339	0.99	1587	0.23	1699	1.02	0.23
SCS-3:1	1	1341	0.99	1593	0.2	2779	1.01	0.2
SGF-5:3	1	1346	0.98	1583	0.26	2699	1.02	0.26
SCS-5:3	1	1341	1	1598	0.19	2755	1	0.19

## Data Availability

The data presented in this study are available on request from the corresponding author.
